# Supplementary material for: Cross-cultural adaptation of the 5-Question Stigma Indicators in trachoma-affected communities, Ethiopia
Source: PLOS Ment Health. 2024 Nov 27;1(6):e0000191. doi: 10.1371/journal.pmen.0000191 (PMC7616881; doi:10.1371/journal.pmen.0000191)
Supplement: S1 Table — (DOCX) [file pmen.0000191.s002.docx]

**S2 Table: Social distance scale English version**

**Female**

Please read the following statement **(vignette)** to the participant.

Abebech is 27-year-old young women. She is a framer, and like most people in her village, she gets sufficient harvests from her farm every year. However, she has problem on both eyes. Her eyes continuously water, have discharges and are painful. Her eyelashes are rotated towards her eyes and are constantly scratching her eyes. Her eye vision is reducing gradually. She is not able to open her eyes completely as doing so worsen the pain and is afraid of the light. Recently, health workers visiting her village has told her that she has trachoma related eye problem that can lead to blindness and that she need to be treated surgically. Abebech wants to meet a man and get married and have a family. However, she is always worried about her eye condition.

Please, read each sentence and the answer choices to the participant and ask her to select the answer that best fits her opinion.

| SN | Questions | Definitely willing | Probably willing | Probably not willing | Definitely not willing | Score |
| --- | --- | --- | --- | --- | --- | --- |
| 1 | How would you feel about renting a room in your home to someone like Abebech? | 0 | 1 | 2 | 3 |  |
| 2 | How about working in the same job with someone like Abebech? |  |  |  |  |  |
| 3 | How would you feel having someone like Abebech as a neighbour? |  |  |  |  |  |
| 4 | How about having Abebech as caretaker of your children? |  |  |  |  |  |
| 5 | How about having one of your children marry someone like Abebech? |  |  |  |  |  |
| 6 | How would you feel about introducing Abebbech to a young man you are friendly with? |  |  |  |  |  |
| 7 | How would you feel about inviting Abebech to social gatherings (such as weddings)/ holiday events? |  |  |  |  |  |

**Male**

Please read the following statement **(vignette)** to the participant.

"Abebe is a 35-year-old Man. He is a framer, and like most people in his village, he gets sufficient harvests from his farm every year. However, he has problem on both eyes. His eyes continuously water, have discharges and are painful. His eyelashes are rotated towards his eyes and are constantly scratching his eyes. His eye vision is reducing gradually. He is not able to open his eyes completely as doing so worsen the pain and is afraid of the light. Recently, health workers visiting his village has told him that he has trachoma related eye problem that can lead to blindness and that he need to be treated surgically. Abebe wants to meet a woman of his age, get married and have a family. However, he is always worried about his eye condition."

Please, read each sentence and the answer choices to the participant and ask him to select the answer that best fits her opinion.

| SN | Questions | Definitely willing | Probably willing | Probably not willing | Definitely not willing | Score |
| --- | --- | --- | --- | --- | --- | --- |
| 1 | How would you feel about renting a room in your home to someone like Abebe? | 0 | 1 | 2 | 3 |  |
| 2 | How about working in the same job with someone like Abebe? |  |  |  |  |  |
| 3 | How would you feel having someone like Abebe as a neighbour? |  |  |  |  |  |
| 4 | How about having Abebe as caretaker of your children? |  |  |  |  |  |
| 5 | How about having one of your children marry someone like Abebe? |  |  |  |  |  |
| 6 | How would you feel about introducing Abebe to a young woman you are friendly with? |  |  |  |  |  |
| 7 | How would you feel about inviting Abebe to social gatherings (such as weddings)/ holiday events? |  |  |  |  |  |
