# Supplementary material for: Cross-cultural adaptation of the 5-Question Stigma Indicators in trachoma-affected communities, Ethiopia
Source: PLOS Ment Health. 2024 Nov 27;1(6):e0000191. doi: 10.1371/journal.pmen.0000191 (PMC7616881; doi:10.1371/journal.pmen.0000191)
Supplement: S2 Table — (DOCX) [file pmen.0000191.s003.docx]

**S3 Table: Social distance scale Amharic version**

**Female**

**የማህበራዊ ርቀት መለኪያ (ለሴት)**

እባኮትን የሚከተለውን መግለጫ (ቪግኔት) ለተሳታፊው ያንብቡ::

አበበች የ 27 ዓመት ወጣት ናት፡፡ በግብርና የምትተዳደር ስትሆን ከቤተሰቧ ጋር በመሆን በዓመት እንደማንኛውም የአካባቢው ነዋሪ በግብርና በቂ የሚባል ምርት ታገኛለች፡፡ ሆኖም ሁለቱንም ዓይኗን ያማታል፡፡ ዓይኖቿ ያለማቋረጥ ያለቅሳሉ፣ የሳሻታል እንዲሁም ከፍተኛ ቅምጥ እና የዓይን ፈሳሽ ይይዛል፡፡ የዓይን ሽፋሽፍቷ ወደ ዓይኗ ውስጥ ተቀልብሶ ያለማቋረጥ እንደ ጦር እንደሚወጋት ትናገራለች፡፡ የማየት አቅሟም ቀስ በቀስ እየቀነሰ ነው፡፡ ዓይኗ ብርሀን ሰለሚፈራ እና የህመም ስሜቱ ስለሚባባስባት ዓይኗን ሙሉ በሙሉ ለመግለጥ ትቸገራለች፡፡ አሁን አሁን የዓይን ህመሟ የዕለት ስራዋን በአግባቡ እንዳታከናውን እየገደባት ነው፡፡ በቅርቡ ወደ መንደሯ የመጡ የጤና ባለሙያዎች ዓይኗን መርምረው በትራኮማ ምክንያት የሚመጣ ለዓይነ-ስውርነት የሚዳርግ የዓይን ሽፋሽፍት ወደ ዓይን ውስጥ መቀልበስ ችግር እንዳለባት እና በቀዶ-ህክምና መታከም እንዳለባት ነግረዋታል፡፡ አበበች በቅርቡ ማግባት እና ቤተሰብ መመስረት ትፈልጋለች፡፡ ከሰዎች ጋር ያላት ግንኙነት መልካም የሚባል ነው ነገር ግን ሁልጊዜም የዓይኗ ጉዳይ ያሳስባታል፡፡

እባኮትን እያንዳንዱን ዓረፍተ ነገር እና የመልስ ምርጫዎችን ለተሳታፊው አንብቡ እና ለእሷ አመለካከት የሚስማማውን መልስ እንድትመርጥ ጠይቋት::

| ተ.ቁ | ጥያቄ | በርግጠኝነት ፈቃደኛ ነኝ | ምንአልባት ፈቃደኛ እሆናለሁ | ምንአልባት ፈቃደኛ አልሆንም | በርግጠኝነት ፈቃደኛ አይደለሁም | ነጥብ |
| --- | --- | --- | --- | --- | --- | --- |
| 1 | ለአበበች በቤትዎ ከፍል ለማከራየት ምን ያህል ፈቃደኛ ነዎት? | 0 | 1 | 2 | 3 |  |
| 2 | ከአበበች ጋር አንድ ላይ ስራ ለመስራት ምን ያህል ፈቃደኛ ነዎት? | 0 | 1 | 2 | 3 |  |
| 3 | አበበች የእርስዎ ጐረቤት ብትሆን ምን ያህል ፈቃደኛ ነዎት? | 0 | 1 | 2 | 3 |  |
| 4 | አበበች የእርስዎን ልጆች እንድንትከባከብ ምን ያህል ፈቃደኛ ነዎት? | 0 | 1 | 2 | 3 |  |
| 5 | አበበች ልጅዎትን እንድታገባ ምን ያህል ፈቃደኛ ነዎት? | 0 | 1 | 2 | 3 |  |
| 6 | አበበችን ከእርስዎ ጋር ቅርበት/ግንኙነት ያለው ሰው ጋር ለማሰተዋወቅ ምን ያህል ፈቃደኛ ነዎት? | 0 | 1 | 2 | 3 |  |
| 7 | አበበች በማህበራዊ ግንኙነቶች (እንደ ሰርግ፣ ጠበል፣ ከርስትና) ወይንም ክብረ በዓል ዝግጅት ላይ ከእርስዎ ጋር እንድትሣተፍ ለመጋበዝ ምን ያህል ፈቃደኛ ነዎት? | 0 | 1 | 2 | 3 |  |

**Male**

**የማህበራዊ ርቀት መለኪያ (ለወንድ)**

እባኮትን የሚከተለውን መግለጫ (ቪግኔት) ለተሳታፊው ያንብቡ::

አበበ የ 35 ዓመት ወጣት ነው፡፡ በግብርና የሚተዳደር ሲሆን በዓመት እንደማንኛውም የአካባቢው ነዋሪ በግብርና በቂ የሚባል ምርት ያገኛል፡፡ ሆኖም ሁለቱንም ዓይኑን ያመዋል፡፡ ዓይኖቹ ያለማቋረጥ ያለቅሳሉ፣ የሳሸዋል እንዲሁም ከፍተኛ ቅምጥ እና የዓይን ፈሳሽ ይይዛል፡፡ የዓይን ሽፋሽፍቱ ወደ አይኑ ውስጥ ተቀልብሶ ያለማቋረጥ እንደ ጦር እንደሚወጋው ይናገራለ፡፡ የማየት አቅሙም ቀስ በቀስ እየቀነሰ ነው፡፡ ዓይኑ ብርሀን ሰለሚፈራ እና የህመም ስሜቱ ስለሚባባስበት ዓይኑን ሙሉ በሙሉ ለመግለጥ ይቸገራል፡፡ አሁን አሁን የዓይን ህመሙ የዕለት ስራውን በአግባቡ እንዳያከናውን እየገደበው ነው፡፡ በቅርቡ ወደ መንደሩ የመጡ የጤና ባለሙያዎች ዓይኑን መርምረው በትራኮማ ምክንያት የሚመጣ ለዓይነ-ስውርነት የሚዳርግ የዓይን ሽፋሽፍት ወደ ዓይን ውስጥ መቀልበስ ችግር እንዳለበት እና በቀዶ-ህክምና መታከም እንዳለበት ነግረውታል፡፡ አበበ በቅርቡ ማግባት እና ቤተሰብ መመስረት ይፈልጋል፡፡ ከሰዎች ጋር ያለው ግንኙነት መልካም የሚባል ነው ነገር ግን ሁልጊዜም የዓይኑ ጉዳይ ያሳስበዋል፡፡

እባኮትን እያንዳንዱን ዓረፍተ ነገር እና የመልስ ምርጫዎችን ለተሳታፊው ያንብቡ እና እሱ አመለካከት የሚስማማውን መልስ እንዲመርጥ ይጠይቁት::

| ተ.ቁ | ጥያቄ | በርግጠኝነት ፈቃደኛ ነኝ | ምንአልባት ፈቃደኛ አሆናለሁ | ምንአልባት ፈቃደኛ አልሆንም | በርግጠኝነት ፈቃደኛ አይደለሁም | ነጥብ |
| --- | --- | --- | --- | --- | --- | --- |
| 1 | ለአበበ በቤትዎ ከፍል ለማከራየት ምን ያህል ፈቃደኛ ነዎት? | 0 | 1 | 2 | 3 |  |
| 2 | ከአበበ ጋር አንድ ላይ ስራ ለመስራት ምን ያህል ፈቃደኛ ነዎት? | 0 | 1 | 2 | 3 |  |
| 3 | አበበ የእርስዎ ጐረቤት ቢሆን ምን ያህል ፈቃደኛ ነዎት? | 0 | 1 | 2 | 3 |  |
| 4 | አበበ የእርስዎን ልጆች እዲንከባከብ ምን ያህል ፈቃደኛ ነዎት? | 0 | 1 | 2 | 3 |  |
| 5 | አበበ ልጅዎትን እንዲያገባ ምን ያህል ፈቃደኛ ነዎት? | 0 | 1 | 2 | 3 |  |
| 6 | አበበን ከእርስዎ ጋር ቅርበት/ግንኙነት ያላት ሴት ጋር ለማሰተዋወቅ ምን ያህል ፈቃደኛ ነዎት? | 0 | 1 | 2 | 3 |  |
| 7 | አበበ በማህበራዊ ግንኙነቶች (እንደ ሰርግ፣ ጠበል፣ ከርስትና) ወይንም ክብረ በዓል ዝግጅት ላይ ከእርስዎ ጋር እንዲሣተፍ ለመጋበዝ ምን ያህል ፈቃደኛ ነዎት? | 0 | 1 | 2 | 3 |  |
