# Supplementary material for: Cross-cultural adaptation of the 5-Question Stigma Indicators in trachoma-affected communities, Ethiopia
Source: PLOS Ment Health. 2024 Nov 27;1(6):e0000191. doi: 10.1371/journal.pmen.0000191 (PMC7616881; doi:10.1371/journal.pmen.0000191)
Supplement: S3 Table — (DOCX) [file pmen.0000191.s004.docx]

**S4 Table: Relevance, and forward and backward translation sufficiency rating guide**

1. **Content validity rating for item relevance**

| 0 | Not relevant (Please insert why in the comment column; recommendation = Drop) |
| --- | --- |
| 1 | Somewhat relevant (Please insert why in the comment column; recommendation = Discuss in the expert panel and decide on whether to keep or drop based on translation quality: recommendation = Keep if adequate, or drop if questionable or inadequate) |
| 2 | Quite relevant (No comment needed; recommendation = Keep) |
| 3 | Very relevant (No comment needed; recommendation = Keep) |

1. **Cultural equivalence sufficiency (forward translation)**

| ***Forward translation sufficiency/quality assessment shall be based on the following four parameters.*** | |
| --- | --- |
| ***Semantic equivalence:*** | Does the expression of the translated item achieve same literal meaning with the source item?  Do the words mean the same thing?  Are there multiple meanings of the word in the target language that might create different response sets by respondents?  Are there grammatical difficulties/problems in the translation? |
| ***Idiomatic equivalence:*** | Are there idiomatic expressions (expressions that have a meaning contrary to the usual meanings of the words)?  If yes, does the translation achieve translation of idioms in a way that that sounds natural in the target language?  If yes, then has equivalent idiom in the target language been used? |
| ***Experiential equivalence:*** | Does the item capture/express the target populations’ everyday life experience? |
| ***Conceptual equivalence:*** | Does the target item describe the concept in the source language with same meaning across different groups (regardless of gender, age, educational status etc) in the target (study population), and adequately map to the intended purpose of the item? Please note that often words hold different conceptual meanings between cultures and different socio-demographic and economic groups. |
| ***Sufficiency (translation quality) Ratings (Please refer to the four sufficiency parameter definitions above):*** | |
| ***1*** | Inadequate (needs detailed special review, describe nature of problem) |
| ***2*** | Questionable (needs discussion, describe nature of problem) |
| ***3*** | Adequate (no comment needed) |

1. **Back-translation sufficiency**

| ***Forward translation sufficiency/quality assessment shall be based on the following four parameters.*** | |
| --- | --- |
| ***Semantic equivalence:*** | Does the expression of the translated item achieve same literal meaning with the source item?  Do the words mean the same thing?  Are there multiple meanings of the word in the back translated item that might create different response sets by respondents ( if used in the source population)?  Are there grammatical difficulties/problems in the translation? |
| ***Conceptual equivalence:*** | Do the back-translated items adequately describe the concept in the source language and adequately map to the intended purpose of the item? Please note that often words hold different conceptual meanings between cultures and different socio-demographic and economic groups |
| ***Sufficiency (translation quality) Ratings (Please refer to the two sufficiency parameter definitions above):*** | |
| ***1*** | Inadequate (needs detailed special review, describe nature of problem) |
| ***2*** | Questionable (needs discussion, describe nature of problem) |
| ***3*** | Adequate (no comment needed) |
