# Supplementary material for: Cross-cultural adaptation of the 5-Question Stigma Indicators in trachoma-affected communities, Ethiopia
Source: PLOS Ment Health. 2024 Nov 27;1(6):e0000191. doi: 10.1371/journal.pmen.0000191 (PMC7616881; doi:10.1371/journal.pmen.0000191)
Supplement: Attachment — Submitted filename: Response to Reviewers_26Sep24.docx [file pmen.0000191.s005.docx]

**Response to Editors and Reviewers Comments**

PLOS Mental Health

PMEN-D-24-00313

Cross-cultural adaptation of the 5-question stigma indicators in trachoma-affected communities, Ethiopia

**Journal Requirements:**

**1. We noticed that you used "unpublished data" in the manuscript. We do not allow these references, as the PLOS data access policy requires that all data be either published with the manuscript or made available in a publicly accessible database. Please amend the supplementary material to include the referenced data or remove the references.**

We have now added the 2022 West Gojjam population data that we got from Ethiopia Central Statistics Agency as S1 Appendix.

**2. Please review your reference list to ensure that it is complete and correct. If you have cited papers that have been retracted, please include the rationale for doing so in the manuscript text or remove these references and replace them with relevant current references. Any changes to the reference list should be mentioned in the rebuttal letter that accompanies your revised manuscript. If you need to cite a retracted article, indicate the article’s retracted status in the References list and also include a citation and full reference for the retraction notice.**

Thank you for picking up the issue around referencing. Our referencing had issues as two different EndNote libraries were used by the first and last author. We have also removed few references that are added as a second or third reference to the literature and kept only the key ones. Below are the changes we made regarding referencing:

- Reference # 17 (Valer et al 2015), #18 (Ridd et all 2011), #19 (Kline 2000) are now removed as they don’t add much to the sample size determination justification, which is already adequately described in our primary references by Anthoine et al 2014 (reference # 17 in the revised version), and Terwee et al 2007 (reference # 18 in the revised version),
- Reference #21 (Henrica C. W. de Vet, 2011) is now removed and replaced by Herdman et al 1998.
- Reference #23 (Nunnally 1994) is no longer needed as Streiner 2003 is sufficient as a reference for the sentence in lines 882 – 884.
- Other references such as Frick et al 2001, Streiner et al 2003 have been edited and recited.

**Reviewers' comments:**

Reviewer 1's Comment]

Trachoma, an eye infection caused by Chlamydia trachomatis, is indeed a neglected tropical disease that suffers particularly from stigma. This condition can lead to blindness if not treated properly and is often associated with poor living conditions. Addressing this issue is highly relevant due to its significant impact on affected communities and the need for effective intervention and support.

To improve this article, I recommend considering these observations.

**1- To enhance this article, please provide a clear explanation of how you determined the sample size and ensure this is detailed in the Methods section.**

Please kindly note that there is a sample size estimation section between lines 243 – 258. We agree with the esteemed reviewer that clarity on the sample size estimation will enhance the article. One of the challenges in cultural equivalence studies is the lack of guidance and consensus in the sample size determination. The most commonly used method is to use subject to item ratio based on the minimum required sample for exploratory factor analysis (please see Anthoine et al 2014 [reference # 17]). That is what we exactly used to determine the sample size in this study.

**2- It is essential to clearly specify the sampling method adopted, whether it is probabilistic or non-probabilistic.**

We completely agree with the esteemed reviewer. Three categories of participants were enrolled in this study:

1) The TT cases, which were identified through a community based active screening, which is the most acceptable method to identify TT cases, given that the average prevalence of TT currently even in high endemic setting is about 2% due to the high-volume surgical services provided by trachoma control programmes in the last decade. So, selecting TT cases at random is not an efficient method. Therefore, what we did is do the active community screening until the required sample is reached, which is not a probabilistic sampling method. We have edited the sentence in lines 160 – 161 to insert the non-probable sampling phrase as follows:

*“Persons with TT were identified through active community-based screenings in high-burden districts in non-probability sampling until the required sample size was reached.”*

2) Persons without TT (location-, sex-, and age-matched to the corresponding persons with TT) were selected randomly from the list of people in the village of the TT cases using a lottery method. Therefore, to add clarity that this sampling was probabilistic we have inserted the following sentence in line 176.

*“These were selected in probability sampling technique.”*

3) The experts involved in the conceptual, semantic, and item equivalence measurement, were selected purposively. This is clearly indicated in lines 185 – 186.

**3- You should develop and include the path diagram, incorporating the correlation coefficients, to provide a clearer representation of the study’s framework.**

Thank you for the suggestion. We have clearly indicated the study framework in Figure 1, and the correlation coefficient for the measurement equivalence component of the study are summarised in Table 9. The other components are largely qualitative and cannot be presented in a form of correlation coefficients in a single framework.

[Reviewer 2's Comment]

**I found this study valuable and believed it would contribute to the literature. Elaborating on just a few points would strengthen the article. My suggestions are as follows:**

**- The necessity of the study should be detailed in the introduction section, and its purpose and hypothesis(s) should be stated.**

Thanks for the suggestion. The necessity of the study has been described between lines 74 – 87. Unlike in other studies, in a such cross-cultural adaptation study, there will not be a single hypothesis that can be indicated in the introduction. However, there will be multiple hypotheses that need to be tested within each cultural equivalence testing variables. For instance, to test construct validity we had two predefined hypotheses. These are indicated in lines 353 – 355, and 359 – 361. We have also indicated the null hypothesis for the Bartlett’s test of sphericity in lines 424 – 425. The cut off point for other tests such as KMO and Cronbach’s alpha coefficient have also been indicated in the manuscript, which tap into the null hypothesis.

To add clarity on the purpose of the study and its significance as per the recommendation from the esteemed reviewer, we have now added the following sentence in lines 88 – 93

*“The purpose of this study is to fill this gap by validating a quantitative stigma tool that can help to rapidly measure and monitor stigma associated with TT and the impact that the surgical service provided to treat TT has on stigma. The data generated using adequately validated tools would be used to encourage allocation of resources to address the unmet mental healthcare needs of trachoma and other NTDs affected communities and thereby facilitate and support the elimination of these diseases by 2030.”*

- Other scales related to the subject in the literature should be mentioned and the reason for choosing this scale and its advantages over other scales should be explain.

This is excellent point. We have now edited the introduction to clarify why the 5-QSI scales are selected for this study. We have moved few sentences from the methods section to the introduction to make this point clear early on. The following paragraph has been inserted in lines 95 – 104.

*“There are several stigma measurement tools. The most used in NTD affected communities are Stigma Assessment and Reduction of Impact (SARI, 21 item scale), Explanatory Model Interview Catalogue (EMIC, 15 item scale), the Social Distance Scale (SDS, seven item scale), and the 5-Question Stigma Indicators (5-QSI, 5 item scale) [8]. The 5-QSI not only have the fewest items but also the easiest to administer. The 5-QSI scales are mentioned in the ‘suggested actions’ section of the Global Leprosy Strategy 2016–2020 Monitoring & Evaluation Manual as an easy-to-use questionnaire for stigma assessment [9]. The relative ease of administration makes the 5-QSI ideal tools to measure and monitor stigma in NTDs, raising the need to expand validation in diverse communities and health conditions, which is why it is selected for cross-cultural adaption in this study.”*
